# Notes on the genus *Taridius* Chaudoir, 1875 (Coleoptera, Carabidae, Lebiini), with descriptions of six new species from Vietnam

**DOI:** 10.3897/zookeys.244.3836

**Published:** 2012-11-26

**Authors:** Dmitri N. Fedorenko

**Affiliations:** 1 A. N. Severtsov Institute of Ecology and Evolution, Russian Academy of Sciences, Moscow, Russia

**Keywords:** New species, *Taridius*, Carabidae, Coleoptera, Vietnam

## Abstract

Six new species of the genus *Taridius* Chaudoir, 1875 (Coleoptera, Carabidae) are described from Vietnam: *Taridius ornatus* **sp. n.**, *Taridius piceus* **sp. n.**, *Taridius fasciatus* **sp. n.**, *Taridius abdominalis* **sp. n.**, *Taridius coriaceus* **sp. n.**, and *Taridius disjunctus* **sp. n.**. The generic characters are redescribed, based on fresh material, with a key provided to all congeners.

## Introduction

The genus *Taridius* Chaudoir, 1875, is a member of the subtribe Cimindidina Laporte de Castelnau, 1834, tribe Lebiini. Originally, it was established for a single species to which five more species were added afterwards (Bates 1892, Andrewes 1935, Emden 1937). Kirschenhofer (2010) contributed further five species to the genus, these being new or earlier described by him in the genera *Cymindis* Latreile, 1806, and *Perseus* Kirschenhofer, 2003. He also downgraded the latter genus to a subgenus of *Taridius* and placed there all congeners, leaving the nominate subgenus monobasic. While the genus is widespread in the Oriental realm, its members have been recorded from type or few close localities, implying highly limited species ranges. Yet this does not seem to be the case because the adults of most of the species are winged and fly well.

The species diversity in the genus is unevenly distributed across the realm. In particular, five species have been reported from India, one from Myanmar, another one from Malay Peninsula, three from the Greater Sunda Isles (Java and Borneo), and only one species from Vietnam, Indochina. Several recent expeditions of the Joint Russia-Vietnam Tropical Center to southern Vietnamese provinces have shown that Vietnam actually supports a rich fauna of *Taridius*.

Six new species are described below from material taken during those expeditions, with a key to all congeners being provided to follow those of Andrewes (1935) and Kirschenhofer (2010). I also think it advisable to refine descriptions of the genus, as well as its two species, *Taridius sabahensis* and *Taridius wrasei*, by adding some important characters absent from the original descriptions. Indices used are based on the measurements as follows: total body length between the apices of the mandibles and elytra, length of the elytra from their basal margins to the apices, maximum widths of both the pronotum, elytra and head across eyes, as well as length of the pronotum along its mid-line.

Holotypes and some paratypes are deposited in the Zoological Museum of the Moscow State University, the other paratypes in the author’s reference collection, A.N. Severtsov Institute of Ecology & Evolution, Russian Academy of Sciences (SIEE). A few specimens of *Taridius* have also been studied from the collection of the Moscow Pedagogical State University (MPSU). All the labels are typewritten, type ones also being red. These are uniform as follows: “HOLOTYPE or PARATYPE / Taridius / species name, sp. n. / D. Fedorenko des., 2011”.

## Taxonomy

### Genus Taridius

Chaudoir, 1875

http://species-id.net/wiki/Taridius

#### Type species:

*Taridius opaculus* Chaudoir, 1875, by monotypy.

#### Redescription.

**Figures 1–10.**
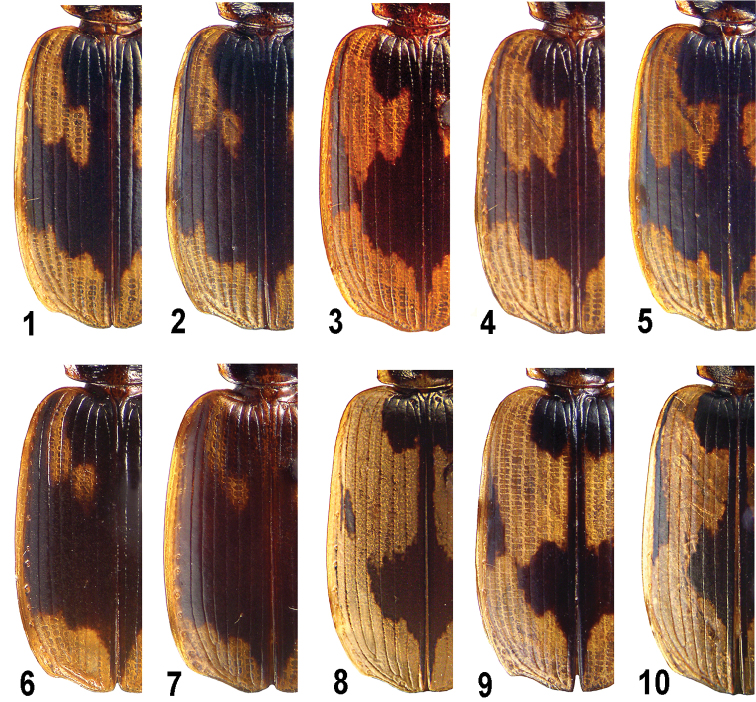
Genus *Taridius*, elytral pattern: *Taridius fasciatus* sp. n. (**1, 2**), *Taridius abdominalis* sp. n. (**3, 4**), *Taridius sabahensis* (**5**), *Taridius ornatus* sp. n. (**6**), *Taridius piceus* sp. n. (**7**), *Taridius disjunctus* sp. n. (**8**), *Taridius wrasei* (**9**), *Taridius coriaceus* sp. n. (**10**).

Body medium-sized, subconvex to flattened. Dorsum glabrous, ventral surface glabrous or sparsely ciliate. Body dark brown to black, mouthparts, clypeus anteriorly, antennae, legs, propleura, reflexed side margin of pronotum, pronotal apex before site of front transverse impression, as well as base behind basal transverse impression mostly pale, yellow. Elytra black or dark brown, usually with a pale, yellow to pale brown, side border, epipleura and an ornate pattern which sometimes bears very slight bronzed reflexions over dark color. This pale pattern (Fig. 1) is generally composed of a large apical spot (PAS) and a humeral vitta (PHS) subdivided into two. Its external part, PHSe, long, running on intervals 6 and 7 and expanded onto 5th behind, while a shorter and smaller internal part, PHSi, occupying intervals 2 to 4 or 3 and 4 before middle. When increasingly extended backwards, PHSe makes a subequally wide, dark, subtransverse, medial fascia (DMF) constricted in interval 6 first (Figs 3–5) and divided into a common (post)median spot (DMS) and a submarginal strip (DLS) after (Figs 8–10). DLS runs on intervals 7 and 8, while tending to be reduced to a small patch at the middle and another one, vague to missing, behind shoulder. The remaining part of the dark pattern, a rounded to quadrate dark spot around scutellum on intervals 1 to 4–5 (DSS) is extended into a sutural strip (DSF) running on intervals 1–2 and tapering behind DMF or DMS. When entire, the dark pattern resembles a flying bird with open wings. Middle of prosternum, meso- and metaventrite mostly pale, reddish or reddish-yellow.

Microsculpture isodiametric on head and elytra, before anterior transverse impression and behind basal transverse impression of pronotum, as well as along its side margin and often also over its more or less wide, posterolateral area. Microsculpture on pronotal disc grated, composed of transverse, very wide and evenly rectangular meshes, rarely these being isodiametric (*Taridius coriaceus* sp. n.). Sometimes longitudinal meshes traceable along side margin between antero- and posterolateral setigerous pores (*Taridius piceus* sp. n.). Elytral microsculpture conspicuous, that on head and pronotal disc often obsolete. Head and pronotum minutely and sparsely punctate.

Eyes convex, genae short to rather long but not or barely projecting, head mostly broadest level to a little before 1/2 length of eye tubercle (eye and gena combined); frontal longitudinal carinae 2–5, rarely seven, on each side. Labrum rectangular, wider than long, very slightly emarginate at and sexsetose along front margin. Submentum bisetose, tooth of mentum stout, rather widely rounded apically, with two setae at middle. Ligula rather wide, truncate, bisetose, paraglossae adnate, slightly surpassing ligula, widely rounded apically. Penultimate labial palpomere plurisetose internally, apical joint fusiform to subtriangular. Antennae filiform, pubescent from antennomere 4 onwards, about last three joints surpassing elytral base, antennomeres 1, 3 and 4 subequally long, 3rd two thirds to nine tenths longer than 2nd; basal three joints with several cilia or very short hairs in addition to standard setae, 2nd mostly with three, preapical, lateral setae anteriorly.

Pronotum bisetose on each side, exceptionally trisetose anteriorly (*Taridius opaculus*), subcordate, broadest far before middle, with front margin slightly sinuate, front angles angles which barely projecting in the latter case. Anterior border interrupted medially, straight or barely sinuate before base; hind angles subrectangular to predominantly obtuse or highly so and rounded. Base oblique towards and often rounded at hind angles which hardly projecting in the latter case. Anterior border interrupted medially, basal border entire, sometimes weaker or interrupted in the middle. Side margin rather widely explanate and strongly reflexed, more so basad, often with more or less dense and large but shallow punctures throughout or only basally. Mid-line fine but moderately deep, extremities excluded, both front and basal transverse impressions weak to indistinct, basal foveae wide, shallow, mostly reduced to a small but rather deep pit just before basal border, giving rise to side gutter, basal transverse impression and a fine to indistinct paralateral line as internal border of basal fovea. A flattened area between this line (or its virtual forward extension) and side border rather densely transversely rugulose, sometimes coriaceous, a sparser and shallower rugosities over disc.

Elytra oblong-oval, humeri rounded, apical truncature slightly oblique, a little sinuate, with outer angle widely or obtusely rounded, apices truncate and mostly sharp, rectangular to slightly obtuse, with an almost indistinct re-entrant angle in the latter case. Angle between side and basal borders (humeral angle) absent or, exceptionally, highly obtuse opposite stria 7, basal border entire and slightly sinuate. Elytral striae moderately deep all along, impunctate or crenulate at base, intervals flat, sometimes (individual variability) odd narrower and a little more convex than even ones. Interval 8 sharply carinate internally in anterior three fourths. Dorsal setigerous pores two, evenly spaced along stria 3, exceptionally (*Taridius nilgiricus* Andrewes, 1935) serial on intervals 3 and 5. Umbilicate series uninterrupted and composed of *ca* 16 setigerous pores. Wings well-developed in all congeners examined but *Taridius piceus* sp. n. Metepisterna long.

Apical margin of last abdominal sternite (VII) bisetose in both sexes but quadrisetose in females of *Taridius disjunctus* sp. n., *Taridius wrasei* Kirschenhofer, 2010 and *Taridius andrewesi* Emden, 1937.

Profemur unisetose in the middle of postero-ventral edge, metacoxa bisetose along outer margin of its posterior part, metatrochanter unisetose, metafemur bisetose; meso- and metatibia dorsally with a more or less distinct, longitudinal sulcus, its edges carinate or at least sharp. Tarsi glabrous dorsally, claw joint setose beneath, claws pectinate. Meso- and metatarsomere 1 as long as the following two combined. Basal three protarsomeres of male dilated and furnished with adhesive vestiture.

Penis (Figs 17–28) in dorsal view mostly with a wide, premedian, swell on right side, apical lamella subtriangular to parallel-sided, widely rounded at tip. Parameres of similar shape in different species (Figs 29–32).

Female gonocoxite IX with two, inner (dorsal) and outer (ventral), ensiform setae varying in size between species. The best developed gonocoxite and setae, especially inner ones, tend to be reduced from *Taridius disjunctus* sp. n. to *Taridius coriaceus* sp. n. (Figs 39–46), inner seta becoming very small and invisible in some species (Figs 43–46).

#### Geographic distribution.

Widespread in South and South-East Asia, from North India and Indochina in the north to Borneo and Java in the south.

#### Habits and habitats.

The beetles occur at the altitudes of 150–1800 m asl where they dwell in leaf-litter of monsoon, broad-leaved, tropical or subtropical forests. Only two specimens of *Taridius ornatus* were taken in coniferous forests, among them a paratype collected in a *Pinus dalatensis* forest using pitfall traps and a female from Chu Yang Sin caught by hands in leaf-litter of an elfin wood under trees of *Chamaecyparis hodginsii* (Dunn, 1908). At higher altitudes, the adults were predominantly taken by pitfall trapping. This is true of *Taridius ornatus* and *Taridius piceus* found to occur syntopically. At low altitudes, beetles mostly flew to lights at night. These were *Taridius fasciatus* and *Taridius abdominalis* taken together, as well as a paratype of *Taridius disjunctus*. A specimen of *Taridius abdominalis* sp. n. was sifted from bamboo leaf litter and individual specimens of the other species (*Taridius coriaceus*, *Taridius disjunctus*, *Taridius wrasei*) were only occasionally collected by hand on the soil surface.

It follows also that up to five species can live sympatrically and at least two syntopically as well. The sympatric species were found to be as follows: (1) *Taridius ornatus*, *Taridius piceus*, *Taridius disjunctus*, *Taridius coriaceus* and *Taridius fasciatus* in the Bi Doup – Nui Ba Nature Reserve, (2) (1) *Taridius ornatus* and *Taridius disjunctus* in the Chu Yang Sin National Park, (3) *Taridius fasciatus* and *Taridius abdominalis* in the Bu Gia Map National Park.

In addition, the fact that different species or their groups show female styli of strongly different structure implies differences in the substrates, soil or leaf-litter of different kind, the larval stages inhabit.

#### Comments.

(1) The congeners are all alike because of a great similarity in body shape. Yet, when combined, the color patterns of both the elytra and the ventral surface of the hindbody are features sufficiently distinctive to discriminate most of the species with certainty.

(2) According to Kirschenhofer (2010), two structural characters, the shape of the last labial palpomere and the number of pronotal anterolateral setae, separate the subgenus *Perseus* from the nominate one which comprises *Taridius opaculus* only. In particular, a spindle-shaped palpomere and a single, standard, seta on the pronotum are peculiar to the former subgenus, whereas a subcylindric palpomere, combined with three anterolateral setae, to the latter. However, the palpomere has been found to be somewhat variable in shape. Firstly, it is subcylindric in males but fusiform in females. Secondly, the apically broadest palpomere is that of *Taridius piceus* sp. n., the species showing only one anterolateral seta on the pronotum. This leaves the polymerous anterolateral setae on the pronotum as the only support to an isolated position of *Taridius opaculus*. Yet, the support is weak because some other characters taken separate, *eg*, the serial dorsal setae on the elytra in the case of *Taridius nilgiricus*, could also provoke the erection of a monobasic subgenus if necessary, polymerous pronotal and elytral setae are deemed hardly different for that purpose. Based on the above evidence, I refrain here from subdividing the genus into the subgenera pending a comparative analysis of male and female genitalia, as well as the number of setae on abdominal sternite VII of the female.

At least two lineages have been traced within the genus. The members of the first lineage share the last abdominal sternite bisetose in both sexes, as well as rather weak female styli supplied with strongly reduced ensiform setae. In addition, the dark elytral pattern is mostly entire, the facial carinae are more numerous, and often also the dorsal microsculpture is superficial on the head and pronotum. In the second lineage which includes *Taridius wrasei*, *Taridius disjunctus* sp. n., *Taridius andrewesi* and probably also *Taridius pahangensis* (Kirschenhofer, 2003), females are distinctive in showing four setae on the last abdominal sternite. A rather pale elytral pattern, conspicuous dorsal microsculpture, less numerous frontal carinae and strongly armed female styli, albeit this feature has been examined in but two species, are among additional characters of the lineage.

#### Key to species of *Taridius*

**Table:** 

1(4)	Elytra monochromous dark, with two discal setigerous pores on interval 3.
2(3)	Small, 6.5 mm in length. Pronotal side margin bisetose, underside black, frons tricarinate on each side at best, elytra short, about three fourths longer than wide. (S-India)	*Taridius niger* Andrewes, 1935
3(2)	Large, 10–10.5 mm in length, anterolateral setae two or three on each side, elytra a half longer than wide, frontal carinae five or more on each side. (N-India, Myanmar)	*Taridius opaculus* Chaudoir, 1875
4(1)	Elytra bicolored, dark with a pale pattern.
5(6)	Elytral discal setigerous pores about half-a-dozen in intervals 3 and 5. Elytral color pattern same as in Fig. 4. (S-India)	*Taridius nilgiricus* Andrewes, 1935
6(5)	Only elytral interval 3 with two discal setigerous pores.
7(24)	Pale spots, PHS and PAS, small, more or less widely separate medially; dark pattern entire (Figs 1–7), rarely with a vague posthumeral stroke detached from.
8(17)	DMF subequally wide, with dark color subequally long on intervals 3 and 6 (Figs 1, 2, 6, 7). Female abdominal sternite VII bisetose.
9(12)	DMF wide due to both pale spots small, PHS narrow, PAS short, transverse, occupying apical fourth of elytra at best.
10(11)	Large, 8.5–9.6 mm in length. Microsculpture very sharp on head and elytra, conspicuous over pronotum, its angles highly obtuse and widely rounded. Mostly two long and one short frontal carina on each side. Elytral dark pattern black, increasingly pale forwards, at base slightly to barely darker than very small PHS (Fig. 7). Humeral angle highly obtuse but distinct. Wingless, elytra wide, 1.38–1.43 as long as wide	2. *Taridius piceus* sp. n.
11(10)	Small, 7.4–8.4 mm in length. Microsculpture obsolete on head and pronotum. Frontal carinae four or five on each side. Elytral dark pattern black throughout, contrastingly darker than pale spots (Fig. 6). No angle between elytral side and basal borders. Winged, elytra longer, 1.44–1.53 as long as wide	1. *Taridius ornatus* sp. n.
12(9)	Either DMF narrower (Figs 1, 2) or pale humeral vitta large, or both.
13(14)	PHSe wide, running on intervals 5 to 8, frontal carinae on each side five. Large, 9 mm in length. (Myanmar)	*Taridius birmanicus* Bates, 1892
14(13)	PHSe narrow, reaching interval 7 only. Body length under 9 mm.
15(16)	Frontal carinae on each side four or five; microsculpture traceable at least along basal and front margins of pronotum. Body small, 6.6–7.5 mm in length	3. *Taridius fasciatus* sp. n.
16(15)	Frontal carinae three at best, body length 7.8–8.0 mm. (N-Vietnam)	*Taridius vietnamensis* (Kirschenhofer, 1996).
17(8)	DMF either reduced to a very narrow lateral process of large DMS between very large PHS and PAS or constricted in interval 6, with dark color 1/4–2/3 or still longer on interval 3 than on 6 (Figs 3–5).
18(19)	PHS and PAS very large, with a very narrow DMF in-between, DLS reduced to a short posthumeral stroke on interval 8. Elytra short, 1.4 times as long as wide. Dorsal microsculpture conspicuous. Underside dark. Frontal carinae three on each side. Body length 7.8 mm. (NE-India)	*Taridius jendeki* Kirschenhofer, 2010
19(18)	PHS and PAS widely separated, DLS well-developed on outer intervals in anterior two thirds or at least in middle third. Elytra about half as long as wide. Abdomen often pale laterally or almost entirely.
20(21)	Abdomen pale throughout but laterally. Elytral dark pattern with very slight bronzed tinge, DLS entire. Frontal carinae four or five on each side. Female abdominal sternite VII bisetose. Body small, 7.2–7.4 mm in length	4. *Taridius abdominalis* sp. n.
21(20)	Abdomen either dark or with anterior two or three sternites pale laterally.
22(23)	Abdomen entirely dark. Frontal carinae four on each side. Female abdominal sternite VII bisetose. Elytral pattern as in Fig. 5, a short posthumeral stroke detached from DLS. Body large, 8.5–9 mm in length. (Borneo)	5. *Taridius sabahensis* (Kirschenhofer, 2003)
23(22)	Anterior two or three abdominal sternites pale laterally. Frontal carinae two or three on each side. Female abdominal sternite VII quadrisetose. Body length 7–8.5 mm. (Java)	*Taridius andrewesi* Emden, 1937
24(7)	PHS and PAS very large and adjoining on interval 6 or also on 5, sometimes vaguely separated, DLS separate or almost so (Figs 8–10). Dorsal microsculpture conspicuous and usually sharp. Very slight bronzed tinge mostly traceable on elytra.
25(30)	Abdomen dark; frontal carinae two or three on each side.
26(29)	DLS long, well-developed in anterior three fourths. Female abdominal sternite VII bisetose.
27(28)	Body small, 7–8 mm in length. Elytral dark pattern without metallic tinge. (India)	*Taridius stevensi* Andrewes, 1923
28(27)	Body large, 8.6 mm in length. Elytral dark pattern with slight bronzed tinge. Pronotum with sharp and dense transverse rugosities over disc but its middle, and a very sharp microsculpture	6. *Taridius coriaceus* sp. n.
29(26)	DLS reduced to a small and oblique spot in third sixth (Fig. 8); slight bronzed reflexions over dark color. Female abdominal sternite VII quadrisetose. Body large, 8.2–8.9 mm in length	7. *Taridius disjunctus* sp. n.
30(25)	Abdominal sternites laterally pale. DLS well-developed in middle third but reduced anteriorly.
31(32)	Frontal carinae mostly two on each side, with an additional and rather slight outer carina before anterior supra-orbital seta. Body length 7.2–8 mm. Female abdominal sternite VII quadrisetose. (Sabah, Borneo)	8. *Taridius wrasei* Kirschenhofer, 2010
32(31)	Frontal carina one on each side. Body length 7.9 mm. (Pahang, Malay Peninsula)	*Taridius pahangensis* (Kirschenhofer, 2003)

### 1. Taridius ornatus

Fedorenko sp. n.

urn:lsid:zoobank.org:act:4717ABE4-61DF-4EC2-873A-2A317259A5D7

http://species-id.net/wiki/Taridius_ornatus

Figs 6 11 20 26 37 38, 43

#### Description.

**Figures 11–12.**
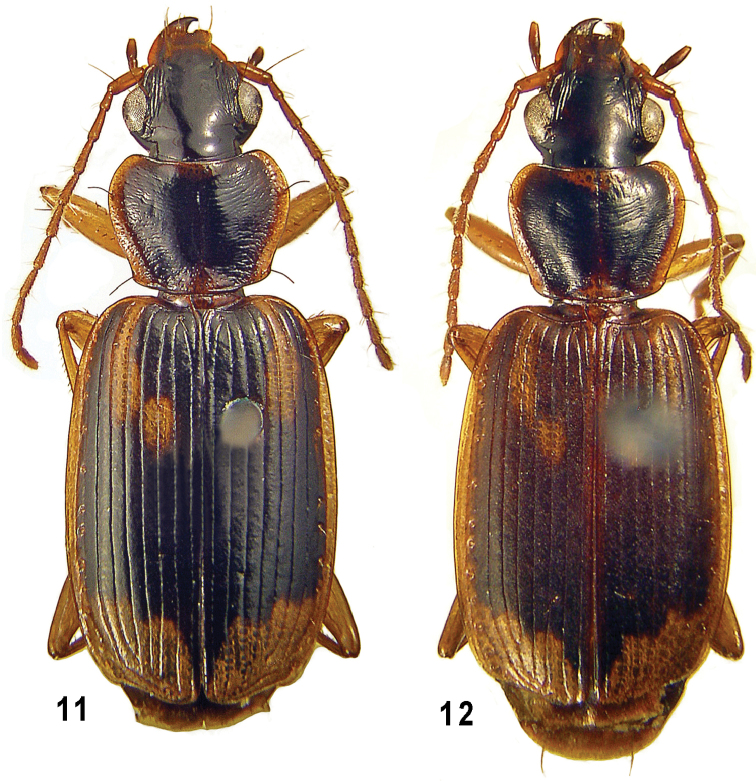
*Taridius ornatus* sp. n. (**11**) and *Taridius piceus* sp. n. (**12**), habitus.

Body (Fig. 11), especially elytra, rather flat, 7.4–8.4 mm in length. DMF very wide (Fig. 6), outwardly extended to interval 9, PAS fairly short and transverse; PHSe short, expanded to interval 5 posteriorly and mostly also to interval 7 anteriorly, PHSi small, occupying intervals 3 and 4 posterior to PHSe, sometimes almost isolated. DSS transverse, mostly subangulate posterolaterally. Underside dark, middle of prosternum, meso- and metaventrite, as well as a narrow apical margin of abdominal sternite VII pale; outer half of metacoxae infuscate. Microsculpture coarse on elytra, obliterate on both pronotum and back half of head, with almost indistinct traces here and there on pronotal disc.

Eyes slightly flattened, genae rather long and a little projecting, head broadest slightly behind 1/3 eye tubercle length; frontal carinae on each side mostly four; of them, inner usually weaker, irregular, shortened at both extremities or only from behind, occasionally obsolete or duplicated. Vertex flattened, neck-constriction indistinct.

Pronotum 1.29–1.39 (mean 1.33, n=8) times as wide as head, 1.33–1.44 (mean 1.39) times as wide as long, base slightly convex backwards, a very small indentation between it and its lateral parts, the latter conspicuously oblique and increasingly curved forwards, sides subsinuate to nearly straight before base, hind angles highly obtuse and often rounded off, except for a small and blunt tooth bearing posterolateral seta. Front transverse impression obsolete or highly superficial, basal transverse impression shallow to moderately deep, slightly deeper at sides, basal foveae wide and shallow, at bottom each usually with a small, deep, transverse pit close to basal border, the latter interrupted or nearly so at middle. Anterior border widely interrupted at middle. Paralateral line obsolete or hardly traceable in basal half only. Coarse submarginal rugosities moderately dense and restricted to basal half. Coarse punctation rather dense over reflexed side margin and adjacent parts of disc in its basal half.

Elytra oblong-oval, flat, 1.44–1.53 (mean 1.49) times as long as wide, 1.45–1.55 (mean 1.5) times as wide as pronotum. Striae impunctate, with almost invisible punctures at bottom, three or four inner striae distinctly shallower within DMF, intervals flat and subequally wide, rarely odd in basal third distinctly narrower than even ones. Last abdominal sternite bisetose along apical margin in both sexes.

Penis as in Figs 20 and 26, apical lamella moderately long, widely rounded at tip. Female gonocoxite IX long and narrow, ensiform setae small, dorsal one invisible in ventral view (Fig. 43).

**Diagnosis.** *Taridius ornatus* is distinguishable from a very similar species, *Taridius vietnamensis* (Kirschenhofer, 1996), in the more extensive dark pattern on the elytra, especially, in a wider and more transverse DMS, flattened eyes, a little projecting genae and rather flat elytra. In addition, the latter species has been taken at much lower altitudes, 620–750 m asl.

#### Variability.

In a female from Chu Yang Sin (see below), the elytral DAS is slightly longer while the inner elytral striae within DMF are less shallow than in the type series.

#### Material.

Holotype ♂: “S[outh] Vietnam, Lam Dong Prov. / Bi Doup – Nui Ba [Nature] Reserve / 12°07'N, 108°39'20"E / Bi Doup Mt., N. slope / h = 1700–1900 m [asl], 16.IV.2008, leg. D Fedorenko”. Paratypes, 5 ♂♂ and 4 ♀♀, taken together with holotype, as well as 12. and 19–22.IV.2008; 1♂, same locality, but env. Long Lanh / 12°10'44"N, 108°40'44"E / h = 1400–1600 m [asl], 29.III–20.IV.2008.

Additional material: ♀, Vietnam, Dak Lak Prov., Chu Yang Sin Natn. Park, 12°23'48"N, 108°20'59"E, Krong Kmar riv., upper flow, h = 1650 m [asl], 30.III–14.IV.2012, leg. D Fedorenko.

#### Geographic distribution.

Known from three localities within Dalat Plateau, Lam Dong and Dak Lak Provinces, Vietnam.

### 2. Taridius piceus

Fedorenko sp. n.

urn:lsid:zoobank.org:act:29EC3746-78D5-4061-BFF7-E2C1E4A56FD0

http://species-id.net/wiki/Taridius_piceus

Figs 7 12 22 28 35 36, 42

#### Description.

Body (Fig. 12) flat, 8.5–9.6 mm in length. Dorsum rather dull due to a sharp microsculpture, with strong sericeous luster on elytra; head and pronotum dark brown to almost black, elytral dark pattern (Fig. 7) dark brown to black posteriorly while increasingly pale both forwards and outwards, thus being barely darker anteriorly than PHS and side border. Elytral color pattern as in *Taridius ornatus* in general, with PHS a little smaller and interval 9 mostly pale. Ventral surface same colored.

Eyes rather small, genae long and distinctly projecting at eye back margin, head broadest level to about 1/3 eye tubercle length; two, long, frontal carinae on each side, an additional, internal, mostly very short, occasionally well-developed, carina present before eyes, sometimes another, external, very fine and short one traceable. Vertex flattened, neck-constriction indistinct or almost so.

Pronotum 1.23–1.32 (mean 1.26, n=8) times as wide as head, 1.3–1.41 (mean 1.35) times as wide as long, base nearly straight, with lateral parts rather strongly oblique and rounded, sides posteriorly subsinuate to straight, hind angles mostly rounded off or barely traceable at posterolateral setigerous pore. Front transverse impression obsolete, basal transverse impression shallow to moderately deep, basal foveae each reduced to a deep pit at its bottom close to basal border, the latter shallower or subinterrupted at middle. Anterior border interrupted medially. Paralateral line shallow but traceable in basal three fourths, starting from basal pit. Rugosities fairly dense but not very sharp, denser over entire area outside paralateral line. Punctation absent but very sparse minute punctures.

Wingless. Elytra flat, mostly reversely ovate, broadest behind middle, 1.38–1.43 (mean 1.4) times as long as wide, 1.52–1.62 (mean 1.58) times as wide as pronotum; with a highly obtuse, almost indistinct humeral angle opposite or just inside stria 7. Striae impunctate, even intervals flat, odd ones mostly slightly convex in basal third or half and often also a little narrower than even. Last abdominal sternite bisetose along apical margin in both sexes.

Penis (Figs 22, 28) regularly convex on right side, apical lamella long, parallel-sided, with a widely rounded tip forming a small capitulum in lateral view. Female gonosubcoxite IX stout, gonocoxite narrow, curved just before apex, dorsal ensiform seta small but distinct in ventral view (Fig. 42).

#### Diagnosis.

The present species is easily recognizable due to a particular combination of large size, flat, wide and wingless body, dull dorsum, an extensive but rather pale dark pattern on the elytra, etc.

#### Material.

Holotype ♂: “S[outh] Vietnam, Lam Dong Prov. / Bi Doup – Nui Ba [Nature] Reserve / 12°07'N, 108°39'20"E / Bi Doup Mt., N. slope / h = 1700–1900 m [asl], 12.IV.2008, leg. D Fedorenko”. Paratypes, 3 ♂♂ and 4 ♀♀, taken together with holotype, as well as 16. and 19–22.IV.2008.

#### Geographic distribution.

Known from type locality only.

### 3. Taridius fasciatus

Fedorenko sp. n.

urn:lsid:zoobank.org:act:6B8F05A4-C0CF-47E1-A53B-FEC20252C2B2

http://species-id.net/wiki/Taridius_fasciatus

Figs 1, 2 13 17 23 31, 32 44

#### Description.

**Figures 13–14.**
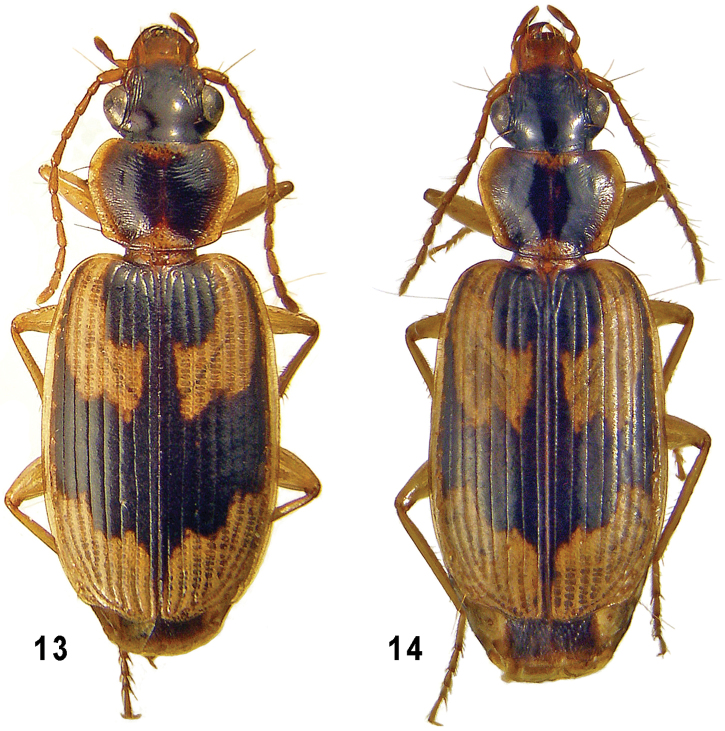
*Taridius fasciatus* sp. n. (**13**) and *Taridius abdominalis* sp. n. (**14**), habitus.

Body (Fig. 13) subconvex and small, 6.6–7.5 mm long. Dorsum black and shining, except for elytra. Pronotal pale pattern rather extensive along anterior and posterior margins. Elytral pale pattern (Figs 1, 2) fairly extensive as well, dark pattern entire, DMF moderately and subequally wide throughout, extended to interval 9 anteriorly and 8 posteriorly, with both front and back margins flexuose; its forward extension along side margin (conformable to DLS) occupying intervals 8 and 9; DSS rounded laterally, on intervals 1 to 5 before while on 1 to 4 behind, mostly angulate posterolaterally, with a straight or concave back margin; one to two inner intervals involved in DSF, often one anteriorly and two just before DMF. PHS entire, PHSi about 1/2 its length posterior to PHSe, the latter extended to interval 7 at humerus. Sometimes PHSe very narrowly separated from PHSi or surpassing it, in the latter case DMF constricted in interval 6. PAS front margin oblique. Ventral surface colored as in previous two species. Microsculpture sharp on elytra, absent from or almost invisible on head, extremely fine on pronotum and obsolete either before its middle or over greater part of disc, posterolateral region excluded; microsculpture a little sharper in female as being very superficial but traceable over head and/or pronotum.

Eyes large and convex, together with genae almost semicircular in outline; frons with 4–5, more often four, carinae on each side, outer ones long, inner not surpassing anterior supraorbital seta; sometimes carinae unilaterally amplified in number to six, irregular, or, otherwise, reduced to three, long ones. Vertex almost flat, neck-constriction barely traceable.

Pronotum 1.19–1.28 (mean 1.25, n=8) times as wide as head, 1.34–1.42 (mean 1.38) times as wide as long, basal margin slightly convex backwards or its almost straight medial part slightly produced, with lateral parts oblique towards and a little rounded at hind angles; anterior margin weakly sinuate, sides subsinuate to straight in basal half, mostly very shortly sinuate just before base, hind angles obtuse or very so, sharp to nearly indistinct. Front transverse impression obliterate, basal transverse impression shallow to moderately deep medially and deeper laterally, thus adjoining or forming a small, more or less deep, transverse pit at bottom of an almost reduced basal fovea, a very fine paralateral line traceable in basal third only. Basal border entire, anterior border very narrow, interrupted at middle. Rugosities rather dense and sharp, especially so posterolaterally, sparser and shallower in the middle of disc. Only reflexed side margin with large, sparse and shallow punctures combined with shorter rugosities.

Elytra flat, oblong-oval to reversely subovate, 1.44–1.57 (mean 1.51) times as long as wide, 1.57–1.71 (mean 1.63) times as wide as pronotum. Striae indistinctly crenulate, intervals subequally wide, flat or barely convex. Last abdominal sternite bisetose along apical margin in both sexes.

Penis as in Figs 17 and 23, its apical lamella subtriangular, more or less widely rounded at tip, the latter slightly curved upwards in lateral view. Female gonocoxite IX (Fig. 44) same as in *Taridius ornatus* sp. n.

#### Diagnosis.

This insect is very similar to *Taridius vietnamensis* and *Taridius ornatus* sp. n., differing from both in the smaller body size and the dorsal microsculpture which is never totally absent from the pronotum. As compared to *Taridius vietnamensis*, the explanate side margin of the pronotum is narrower and less strongly reflexed, with a sharper side gutter, as well as the frontal carinae on each side are four or five instead of three.

Variability. Two additional specimens from higher altitudes (see below) are distinctive in a slightly larger body size and a posteriorly deeper side gutter of the pronotum due to its more strongly reflexed side margin. Furthermore, the male from Thailand shows the pronotum with transverse rugosities denser over the disc, especially along sides, a densely punctate side margin and almost straight lateral parts of the base, as well as the frontal carinae being still more numerous, six or seven, before. Besides this, the apical lamella of the penis is barely curved upwards in lateral view. Yet the endophallus is the same, implying that the differences are intraspecific, subspecific at best.

#### Material.

Holotype ♂: “Vietnam, Binh Phuoc Prov. / Bu Gia Map Nat[ional] Park / 12°11'37"N, 107°12'21"E / h = 540 m [asl] / at light, 15.IV.2009, leg. D Fedorenko”. Paratypes, 20 ♂♂, 19 ♀♀, same data or 17–24. IV.2009.

Additional material: ♀ (MPSU), S-Vietnam, Dak Lak Prov., 75 km N of Phan Tiet, env. Gia Bac, h = 1100 m [asl], 18.IV.2007, leg. P Oudovichenko; ♂ (Collection of P. Bulirsch, Praha), Thailand, Mae Hong Son prov., Kiwlomm-pass near Soppong, 23.6.–2.7.2002, alt. 1400±50 m, WGS 84: 19°26'N, 098°19'E, lgt. Fouquè R. + H. & Kožich J.

#### Geographic distribution.

S-Vietnam (Lam Dong and Dak Lak provinces), Thailand.

### 4. Taridius abdominalis

Fedorenko sp. n.

urn:lsid:zoobank.org:act:ABE5E05A-AB7C-42B0-9FD9-9A25A93FE947

http://species-id.net/wiki/Taridius_abdominalis

Figs 3, 4 14 18 24 29, 30 45

#### Description.

Very similar to the preceding species, except as follows: Body (Fig. 14) on average a little larger, 7.2–7.4 mm in length. Elytral dark pattern as in Figs 3 and 4, DSS with almost indistinct bronzed luster, PHSe long, its back margin level to or behind that of PHSi, DMF strongly constricted in interval 6; DSS rounded, occupying inner 4.5 intervals. Abdomen pale throughout but at sides; often also prosternum, meso- and metaventrites entirely pale. Microsculpture sharper, coarse on elytra, moderately deep over pronotum, superficial but traceable on head.

Frontal lateral carinae mostly four or five, sometimes almost three due to inner carina strongly reduced from behind.

Pronotum 1.23–1.3 (mean 1.26, n=6) times as wide as head, hardly broader, 1.37–1.45 (mean 1.4) times as wide as long; basal margin regularly convex backwards, mostly poorly rounded at hind angles, sides a little or barely sinuate before base, hind angles obtuse but rather sharp. Anterior border interrupted in the middle. Rugosities less sharp. Punctation of base and reflexed side margin often moderately dense, punctures smaller and deeper.

Elytra, 1.5–1.52 times as long as wide, 1.55–1.63 (mean 1.58) times as wide as pronotum. Striae very finely punctate.

Penis (Fig. 24) with very short apical lamella. Female gonocoxite IX almost same as in previous species (Fig. 45).

#### Diagnosis.

This species is distinctive among the others in the combination of elytral and ventral color patterns as described above, an almost entirely pale abdomen being found in no other species.

#### Material.

Holotype ♂: “Vietnam, Binh Phuoc Prov. / Bu Gia Map Nat[ional] Park / 12°11'37"N, 107°12'21"E / h = 540 m [asl] / at light, 17–24. IV.2009, leg. D Fedorenko”. Paratypes, ♂, 3 ♀♀, same data.

Additional material: ♀, (SIEE): S-Vietnam, Dongnai Prov., Nam Cat Tien Natn. Park, *Dipterocarpus* forest, siefted from leaf-litter, 4.XII.2004, leg. A Anichkin.

#### Geographic distribution.

Known from but two localities as above.

### 5. Taridius sabahensis

(Kirschenhofer, 2003)

http://species-id.net/wiki/Taridius_sabahensis

Figs 5 41

Taridius sabahensis Kirschenhofer, 2003: 9 (*Perseus*; Sabah, Borneo).

#### Description.

Body subconvex, 8.5–9 mm long. Dorsum black, moderately shining. Elytral dark pattern (Fig. 5) entire, but for a small and vague posthumeral stroke separated from DLS; DSS subrectangular, expanded onto interval 5 anteriorly and 4 posteriorly, with almost straight back margin, DSF running on interval 1 and barely expanded onto 2nd, DMF constricted on intervals 3 and 6, black color on 6th about two thirds longer than on 3rd, DLS occupying intervals 7 and 8 medially while only 8th anteriorly, terminating a fourth from base and two fifth from apex. Underside colored same as in *Taridius ornatus*. Microsculpture sharp on elytra, moderately sharp over pronotum, less conspicuous on head, very superficial on neck.

Eyes together with fairly long genae rather large, semicircular in outline; four fairly long frontal carinae present. Vertex slightly convex, neck-constriction very weak.

Pronotum 1.36–1.37 times as wide as head, 1.45–1.47 times as wide as long, basal margin a little convex backwards medially, lateral parts very slightly before, oblique forwards and increasingly curved outwards; sides barely sinuate before base, hind angles highly obtuse and almost indistinct; reflexed side margin widely explanate. Front transverse impression obliterate, basal transverse impression rather shallow, basal foveae large but nearly indistinct, each with a small oblique pit at bottom, paralateral line almost indistinct. Basal border entire, anterior border interrupted medially. Disc smooth with very sparse and weak transverse rugosities, very sparse punctures traceable only in basal foveae and close to side gutter just before them.

Elytra rather convex, 1.48–1.51 times as long as wide, 1.44–1.48 times as wide as pronotum, apices truncate and a little obtuse. Striae almost indistinctly crenulate, intervals subequally wide and flat. Last abdominal sternite bisetose in female.

Female gonocoxite IX narrow, ensiform setae moderately developed, dorsal seta distinct in ventral view (Fig. 41)

#### Material.

♀ (MPSU), E-Malaysia, Sabah, Mt. Kinabalu, Natn. Park, 1700 m asl, 16-30.07.2002, leg. Kurbatov & Zimina.

#### Geographic distribution.

Known from a few localities in Sabah (Kirschenhofer 2010).

### 6. Taridius coriaceus

Fedorenko sp. n.

urn:lsid:zoobank.org:act:7CBAC243-A3D9-40CB-B75C-339CBF4D9CA8

http://species-id.net/wiki/Taridius_coriaceus

Figs 10 15 46

#### Description.

**Figures 15–16.**
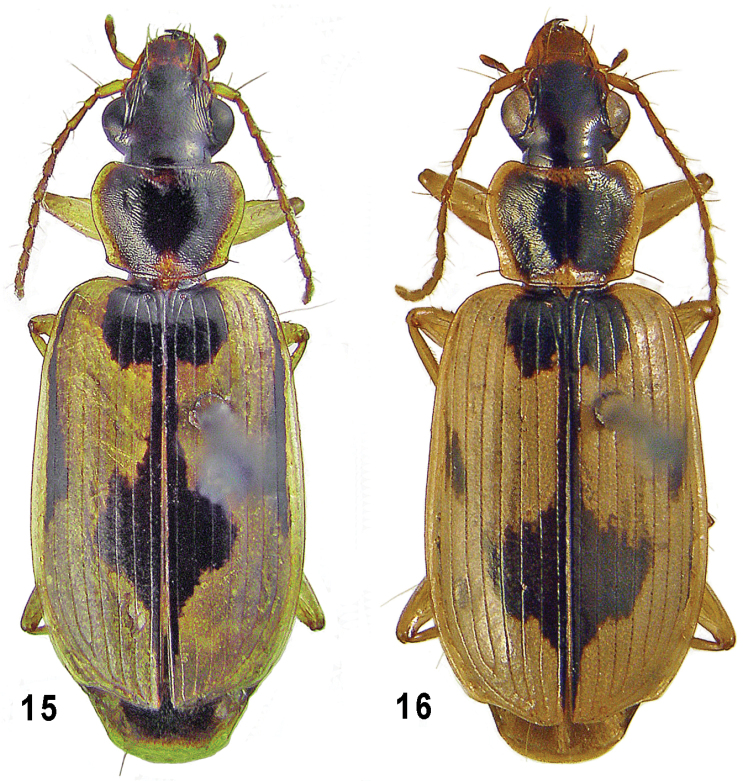
*Taridius coriaceus* sp. n. (**15**) and *Taridius disjunctus* sp. n. (**16**), habitus.

Body (Fig. 15) subconvex, 8.6 mm in length. Dorsum black, a little shining due to a rather sharp microsculpture. Elytral dark pattern strongly reduced (Fig. 10): DSS occupying inner four intervals, slightly concave at back margin, sides and posterolateral angles both rounded, DSF occupying only sutural interval, DMS extended outwards as far as middle of interval 5, DLS separate on interval 8 in anterior three fifths, expanded onto 7th at middle and a little before. Underside black, metaventrite widely yellow medially, mesoventrite reddish at the extreme apex, pro-sternal process hardly paler between coxae, outer half of metacoxa infuscate, abdomen black to dark brown, apical third of its last sternite yellow. Microsculpture sharp on elytra, very sharp, nearly granulate over pronotum, including reflexed side margin, moderately deep, isodiametric or barely transverse, in the middle of disc, very distinct on head, superficial to obsolete on neck.

Eyes and genae combined almost semicircular in outline; frons on each side with three or four, anteriorly with four or five, carinae. Vertex almost flat, neck-constriction barely traceable.

Pronotum 1.17 times as wide as head, 1.39 times as wide as long, same as in as in

*Taridius abdominalis*, but lateral parts of base nearly straight, sides distinctly sinuate before base, hind angles obtuse but rather sharp. Both front and basal transverse impressions, as well as basal foveae except at the very base, obsolete; paralateral line indistinct. A wide area between reflexed side margin and middle of disc coriaceous and dull from irregular, moderately deep and very dense rugosities accompanied by very sharp microsculpture in addition.

Elytra slightly convex, subovate, 1.56 times as long as wide, 1.79 times as wide as pronotum, broadest far behind middle, apices truncate and subrectangular. Striae impunctate, intervals subequally wide and almost flat. Last abdominal sternite bisetose along posterior margin.

Female gonocoxite IX long, parallel-sided, widely rounded apically, ensiform setae almost totally reduced, dorsal setae invisible in ventral view (Fig. 46).

#### Diagnosis.

The species is certain to belong to the group which members share the last abdominal sternite bisetose in both sexes. From all of them, it differs by separate, dark, elytral DLS, peculiar pronotal sculpture and microsculpture, as well as by a particular structure of the female gonocoxite IX. A similar elytral pattern is observed in *Taridius stevensi* which is in contrast smaller and devoid of bronzed luster over dark color. Well-developed DLS and a dark abdomen are uncharacteristic of *Taridius wrasei* and *Taridius pahangensis*, the former species showing a quadrisetose abdominal sternite VII. The latter, according to its original description, is distinctive in having only one frontal carina on each side and a transverse pronotal microsculpture.

#### Material.

Holotype ♀, S[outh] Vietnam, Lam Dong Prov. / Bi Doup – Nui Ba [Nature] Reserve / env. Long Lanh / 12°10'44"N, 108°40'44"E / h = 1400–1600 m [asl], V.2009 / leg. D Fedorenko.

#### Geographic distribution.

Known from type locality only.

### 7. Taridius disjunctus

Fedorenko sp. n.

urn:lsid:zoobank.org:act:ACD9C0D6-0176-448B-BDD8-9EEC25080770

http://species-id.net/wiki/Taridius_disjunctus

Figs 8 16 21 27 39

#### Description.

Body (Fig. 16) subconvex, 8.2–8.9 mm in length. Dorsum black, moderately shining. Elytral dark pattern (Fig. 8) with slight but distinct bronzed luster, strongly reduced laterally: DSS rounded, with inner four intervals involved in, emarginate at DSF which running on sutural interval only, DMS expanded outwards as far as stria 5, DLS reduced to a very small median patch on interval 7 or also on 8. Underside colored as in *Taridius ornatus*. Microsculpture sharp on elytra, moderately sharp over pronotum and head, superficial to obsolete on neck.

Eyes slightly reduced, genae rather long and oblique but not projecting, head broadest in anterior third of eye tubercle length; frontal carinae mostly three, among them inner one shorter and shallower anteriorly. Vertex rather convex, neck-constriction distinct.

Pronotum 1.16–1.23 (mean 1.19, n=3) times as wide as head, 1.31–1.36 (mean 1.33) times as wide as long, basal margin a little convex backwards or its straight medial part slightly surpassing lateral parts, these oblique and increasingly curved forwards; sides sinuate or barely so before obtuse or highly obtuse hind angles. Front transverse impression obliterate, basal transverse impression rather deep, wide, deeper laterally, forming a rather deep, oblique pit at bottom of fairly large basal fovea which is separated from disc by a paralateral line traceable in basal half to two thirds. Both basal and anterior borders entire. Transverse rugosities rather dense and sharp, more so in basal foveae. Punctation invisible or very sparse along reflexed side margin only.

Elytra rather convex, 1.47–1.55 (mean 1.52) times as long as wide, 1.77–1.89 (mean 1.84) times as wide as pronotum, apices truncate and a little obtuse; apical truncature conspicuously sinuate before rather distinct though obtusely rounded outer angles. Striae indistinctly crenulate, intervals subequally wide and almost flat. Last abdominal sternite quadrisetose in female.

Penis (Figs 21, 27) with a very large, triangular, apical lamella (the penis is poorly sclerotized because of an immature condition of the only male examined). Female gonocoxite and gonosubcoxite IX stout, the former with strong ensiform setae (Fig. 39).

#### Diagnosis.

This species is close to *Taridius wrasei* and perhaps also *Taridius pahangensis*, with which it shares such characters as similar body shape and elytral color pattern, as well as a conspicuous dorsal microsculpture. Yet it differs from both in the larger body, entirely black abdomen and strongly reduced, almost indistinct, DLS, from the former also in the smaller eyes with oblique genae, the more convex elytra and a particular structure of both male and female genitalia.

#### Material.

Holotype ♀, S[outh] Vietnam, Lam Dong Prov. / Bi Doup – Nui Ba [Nature] Reserve / 12°07'N, 108°39'20"E / Bi Doup Mt., N. slope / h = 1700–1900 m [asl], 10.IV.2008 / leg. D Fedorenko. Paratypes, ♂, same locality, but env. Long Lanh, 12°10'44"N, 108°40'44"E / h = 1400–1600 m [asl], V.2009; ♀, Vietnam, Dak Lak Prov. / Chu Yang Sin Natn. Park / 12°23'48"N, 108°20'59"E, Krong Kmar riv., upper flow / h = 1000 m [asl], at light, 30.III–14.IV.2012 / leg. D Fedorenko.

#### Geographic distribution.

Lam Dong and Dak Lak provinces, Vietnam.

### 8. Taridius wrasei

Kirschenhofer, 2010

http://species-id.net/wiki/Taridius_wrasei

Figs 9 19 25 33, 34 40

Taridius wrasei Kirschenhofer, 2010: 25 (Sabah, Borneo).

#### Description.

Similar to the previous species, except for as follows: body smaller, 7.2–8 mm in length. No bronzed luster over elytral dark pattern, DSS rounded and occupying intervals 1 to 4 and usually also expanded onto 5th, DLS much larger, triangular, on intervals 7 and 8, terminating one forth and three fifths from base and apex, respectively (Fig. 9). Pale color more strongly developed on ventral surface, including metacoxae, metaventrite mesal to the line between mesocoxa and outer tip of metacoxa, as well as sides of first three abdominal sternites. Microsculpture rather sharp throughout.

Eyes larger, together with short genae more convex, semicircular in outline, head broadest level to about middle of eye tubercle length; usually two, long, frontal carinae on each side and a weak outer one. Vertex fairly flat, neck-constriction almost indistinct.

Pronotum 1.2–1.26 (mean 1.24, n=3) times as wide as head, 1.34–1.4 (mean 1.37) times as wide as long, basal margin slightly and regularly convex backwards or with medial part slightly surpassing lateral ones, these being oblique forwards and nearly straight; sides barely to distinctly sinuate before obtuse to subrectangular and rather sharp hind angles. Front transverse impression obliterate or very shallow, basal transverse impression rather shallow as well, a rather deep pit at bottom of fairly large basal fovea, paralateral line hardly traceable in basal third. Basal border entire, anterior border interrupted medially. Transverse rugosities dense and fairly sharp throughout, sparser and shallower only in the middle of disc. Punctation absent from disc and almost so over reflexed side margins.

Elytra rather flat, 1.47–1.53 (mean 1.5) times as long as wide, 1.70–1.77 (mean 1.73) times as wide as pronotum.

Penis as in Figs 19 and 25, apical lamella fairly long, parallel-sided, widely rounded at tip; left paramere with a short and widely rounded apex (Fig. 33). Female gonocoxite and gonosubcoxite IX (Fig. 40) more slender, and with smaller ensiform setae than in the previous species.

#### Material.

♂, ♀ (SIEE), E-Malaysia, Sabah, Mt. Kinabalu, Natn. Park, 1700 m asl, 16–30.07.2002, leg. Kurbatov & Zimina; ♂, same data, but road Kota – Kinabalu – Tambunan, km 52, 1600–1800 m asl (MPSU).

#### Geographic distribution.

Sabah, Borneo.

#### Comments.

According to Kirschenhofer (2010), this species is barely different from *Taridius pahangensis* from Pahang, Malay Peninsula, mainly in a sharper dorsal microsculpture. If so, the latter species may have two or three frontal carinae on each side of the head instead of only one as specified originally.

**Figures 17–22.**
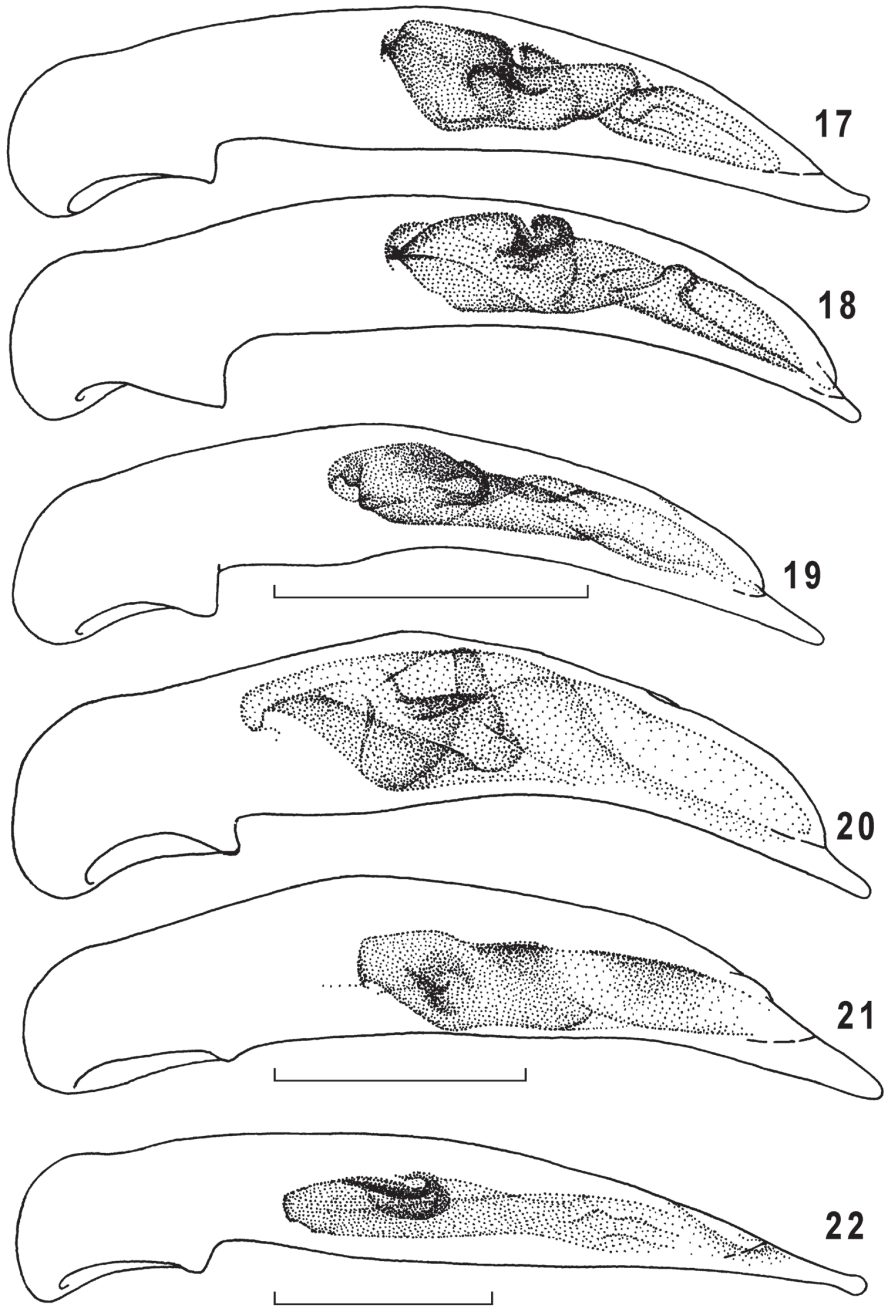
Genus *Taridius*, penis, left lateral aspect: *Taridius fasciatus* sp. n. (**17**), *Taridius abdominalis* sp. n. (**18**), *Taridius wrasei* (**19**), *Taridius ornatus* sp. n. (**20**), *Taridius disjunctus* sp. n. (**21**), *Taridius piceus* sp. n. (**22**); scale bar = 0.5 mm.

**Figures 23–28.**
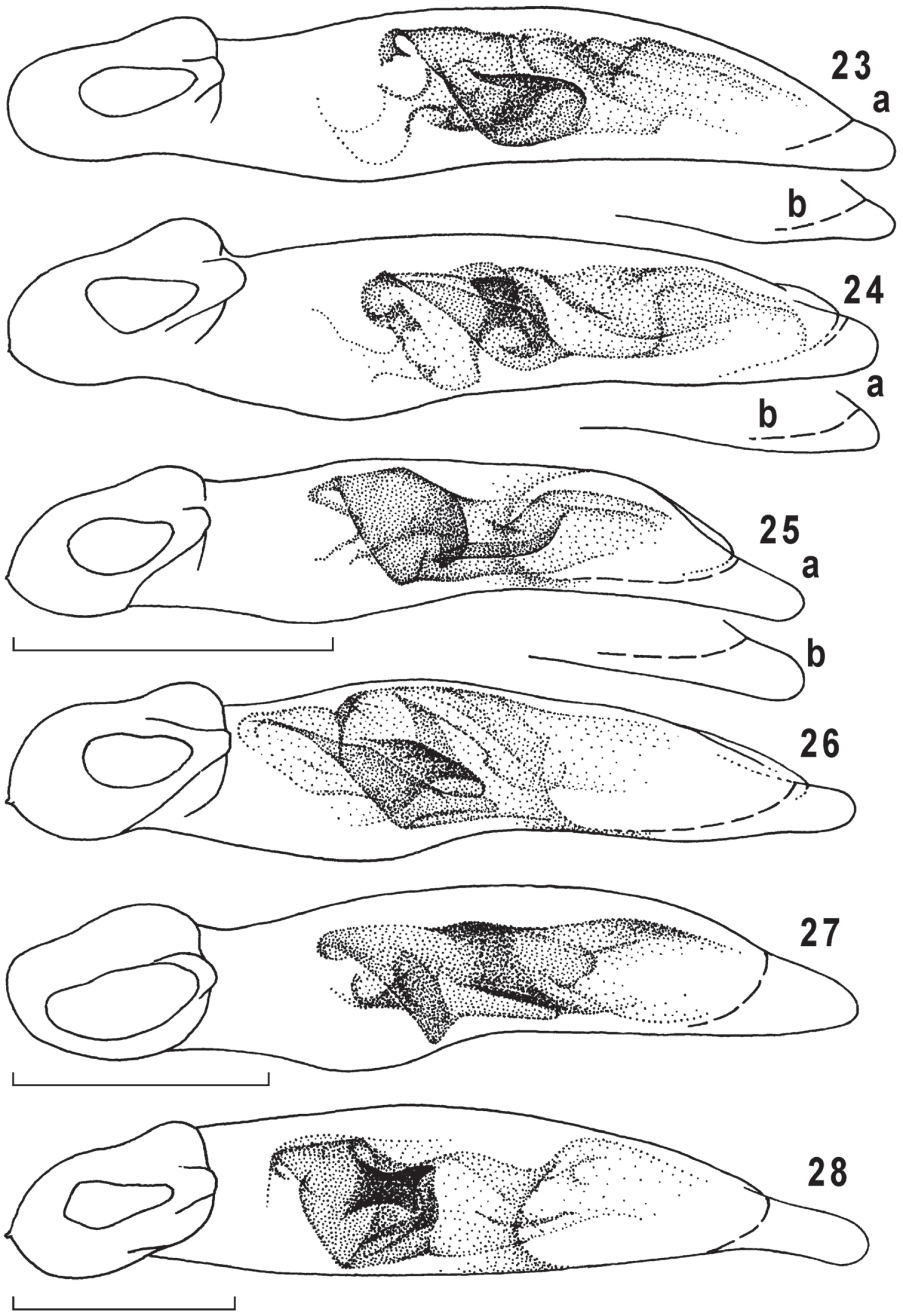
Genus *Taridius*, penis, ventral aspect: *Taridius fasciatus* sp. n. (**23a**), *Taridius abdominalis* sp. n. (**24a**), *Taridius wrasei* (**25a**), *Taridius ornatus* sp. n. (**26**), *Taridius disjunctus* sp. n. (**27**), *Taridius piceus* sp. n. (**28**); variations of penial apex (**b**); scale bar = 0.5 mm.

**Figures 29–38.**
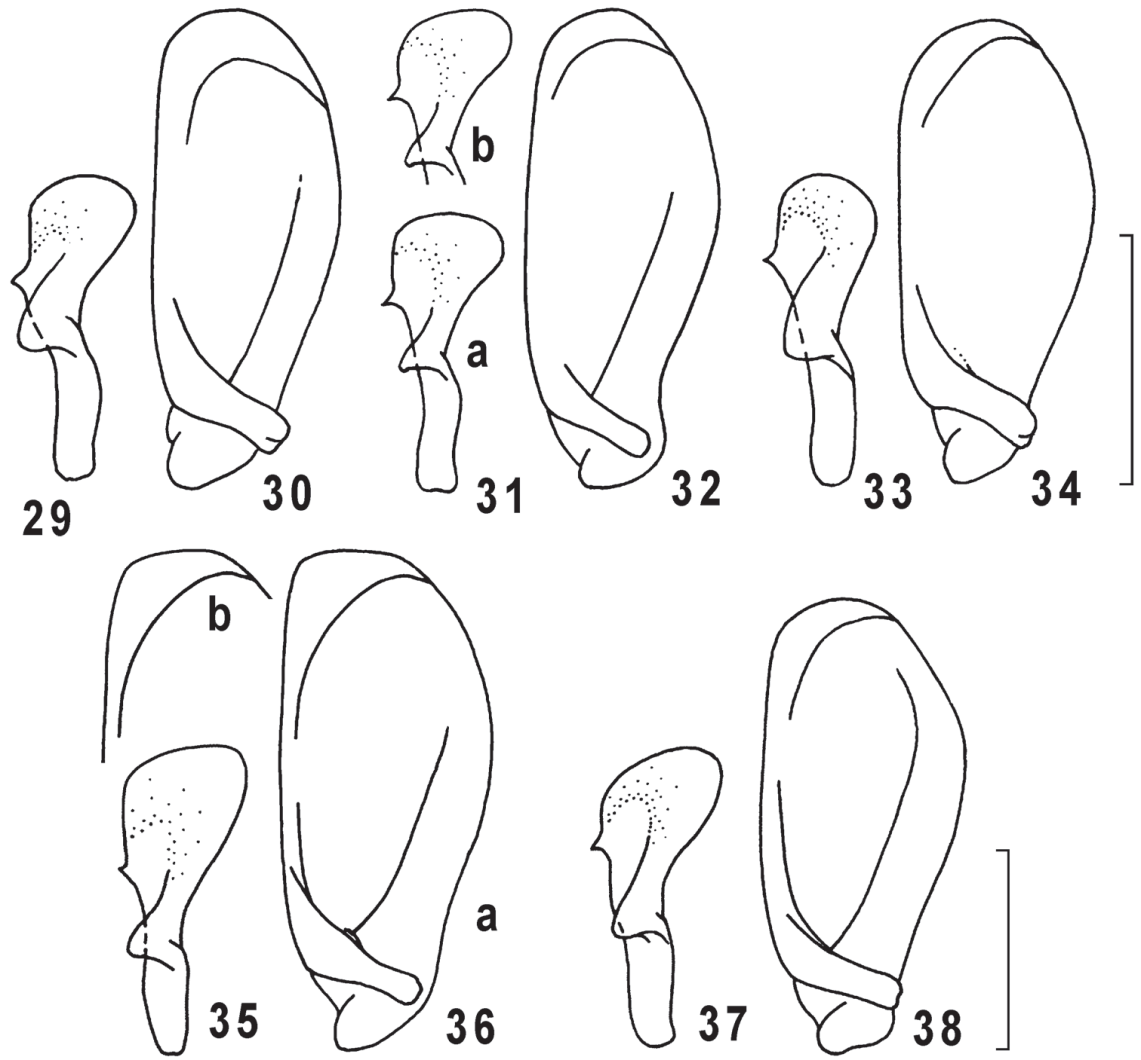
Genus *Taridius*, right (**29, 31, 33, 35, 37**) and left (**30, 32, 34, 36, 38**) parameres: *Taridius abdominalis* sp. n. (**29, 30**), *Taridius fasciatus* sp. n. (**31 a, b, 32**), *Taridius wrasei* (**33, 34**), *Taridius piceus* sp. n. (**35, 36 a, b**), *Taridius ornatus* sp. n. (**37, 38**); scale bar = 0.3 mm.

**Figures 39–46.**
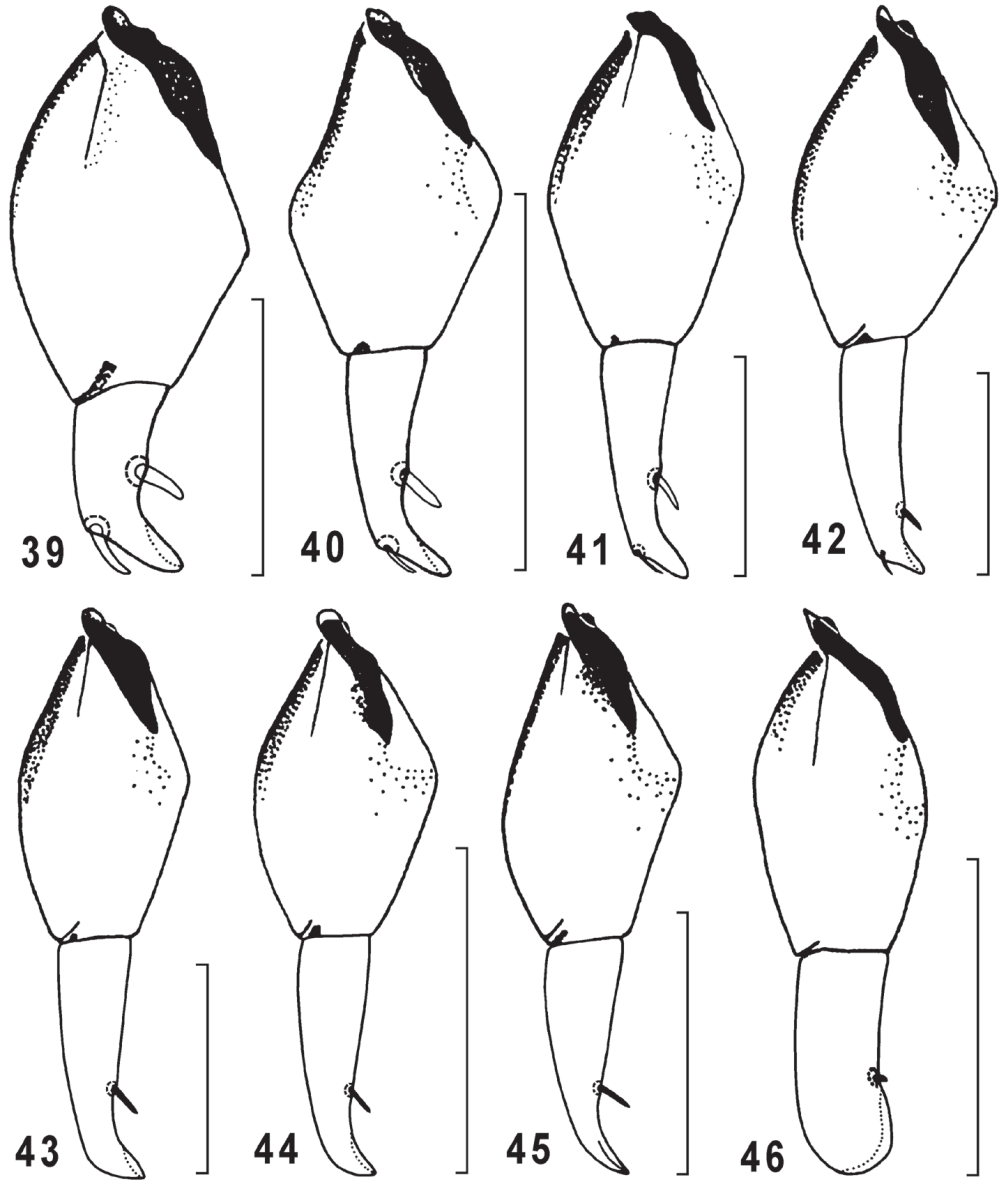
Genus *Taridius*, female left gonocoxite & gonosubcoxite IX, ventral aspect: *Taridius disjunctus* sp. n. (**39**), *Taridius wrasei* (**40**), *Taridius sabahensis* (**41**), *Taridius piceus* sp. n. (**42**), *Taridius ornatus* sp. n. (**43**), *Taridius fasciatus* sp. n. (**44**), *Taridius abdominalis* sp. n. (**45**), *Taridius coriaceus* sp. n. (**46**); scale bar = 0.3 mm.

## Supplementary Material

XML Treatment for Taridius

XML Treatment for Taridius ornatus

XML Treatment for Taridius piceus

XML Treatment for Taridius fasciatus

XML Treatment for Taridius abdominalis

XML Treatment for Taridius sabahensis

XML Treatment for Taridius coriaceus

XML Treatment for Taridius disjunctus

XML Treatment for Taridius wrasei
